# Notes and News

**Published:** 1897-03-13

**Authors:** 


### Notes and News.

(Collected and Communicated.)

HOSPITAL MEETINGS.

National Dental Hospital, Great Portland Street, W.—Annual General Meeting, at the Hospital, on Wednesday, March 31st, at 5 p.m.

Hospital Home for Incurable Children, 2, Maida Vale.—Annual Meeting, at the Hospital, on Saturday, March 13th, at 3 p.m.

A new infirmary has been added to the Bramley Workhouse at a cost of £10,000. The building will contain 80 beds.

Colonel R. Ropner has offered to present Pemberton House at Middleton to the towns of Stockton and Thornby as a convalescent home, if it cannot be obtained through another source. The house will cost £2,000. It will contain 36 patients, and has been used as a convalescent home since 1893.

Unqualified practice appears to have a very large following in America. The law in force against quackery has only been in operation since 1895, yet no less than 51 convictions have been obtained, entailing imprisonment or fines amounting to £738 against the offenders, whilst many other cases are pending.

The exclusive use of sterilised water at the Lure d'Evreux and Chateaudun Barracks, occupied by the French army, has proved so successful that these barracks, previously the frequent scene of typhoid fever, are now reported thoroughly healthy. Sterilising apparatus is now being generally introduced in all military centres in France where the drinking water is not undoubtedly pure.

Mr. James Caird, of Dundee, has offered to erect a hospital for women in the grounds of the infirmary at a cost of £6,000. For this purpose £10,000 had already been raised in donations, and this sum will now be available for maintenance. The new hospital, to be called the Caird Hospital, will consist of two pavilions connected by covered corridors to a central admistration block. The pavilions will contain 13 and 16 beds respectively, with an operating theatre and necessary adjuncts.

The King of Sweden has appointed Mr. P. Michelli, secretary of the Seaman's Hospital, Greenwich, to be a Knight (first class) of the Order of the Wasa.

The Marquess of Bute has promised £5,000 towards the erection of a new hospital for Cardiff, and has also offered a site provided £30,000 is forthcoming from general contributions.

At the last meeting of the Edinburgh Town Council it was decided that a new city hospital for infectious diseases to contain 600 beds should be erected. The cost is estimated at about £233,700.

A new sanitary law has been passed in India, which authorises inspection, and in some cases detention of, travellers by sea and land, and other measures necessary to secure proper sanitary conditions in cases of epidemic.

At last the epidemic of Beri-Beri appears to be abating at the Richmond Lunatic Asylum, Dublin. It is to be hoped that the temporary and later the permanent measures on foot to lessen the overcrowding at the institution will remove all likelihood of the recurrence of the disease, as an epidemic at any rate.

Mr. Colman has purchased and presented to the Jenny Lind Infirmary the site for a new building in memory of his wife. It has been decided to open a subscription list for the building of the new institution, and the Earl of Leicester has headed it with a munificent donation of £2,000.

It is confidently believed that the plague is beginning to die out in Bombay; nevertheless, stringent regulations are now being adopted to prevent the spread of the disease from the city, and in it extension hospitals are being organised, and also segregation camps to receive the occupants of condemned habitations. Calcutta is reported free of the plague.

Some time past we drew attention to the sad plight of Henri Dunant, the founder of the Red Cross Society and one of the most self-denying philanthropists of his day. But into such obscurity had he sunk that the conductors of the very paper which published his poverty were unable to assist us to ascertain his address. We are now glad to hear that his cause has been espoused by the Dowager Empress of Russia, who has made provision for him for the remainder of his days.

A Local Government Board inquiry at the Downpatrick Workhouse Hospital in Ireland has revealed a most unsatisfactory state of things. It appears that matters culminated when two typhoid fever patients were left in the hospital van for three-quarters of an hour, the various persons on duty refusing to remove them, and the master of the workhouse failing to exercise his authority through fear of infection. In the course of the inquiry evidence was given that the mattress on which the patients were placed in the van had not been moved out of it for years, and it was admittedly damp. No provision was made for the regular disinfection of the van, and the fumigating stove was reported out of order. How long this condition of affairs might have continued unchecked it is impossible to say had not the nurses and pauper assistants simultaneously refused to carry the two patients from the van to the wards of the hospital.

